# The Efficacy and Safety of Vitamin C for Iron Supplementation in Adult Patients With Iron Deficiency Anemia: A Randomized Clinical Trial

**DOI:** 10.1001/jamanetworkopen.2020.23644

**Published:** 2020-11-02

**Authors:** Nianyi Li, Guangjie Zhao, Wanling Wu, Mengxue Zhang, Weiyang Liu, Qinfen Chen, Xiaoqin Wang

**Affiliations:** 1 Department of Hematology, Huashan Hospital, Fudan University, Shanghai, China

## Abstract

**Importance:**

It remains uncertain whether vitamin C routinely used with oral iron supplements is essential for patients with iron deficiency anemia (IDA).

**Objective:**

To compare the equivalence and assess the safety of oral iron supplements plus vitamin C or oral iron supplements alone in patients with IDA.

**Design, Setting, and Participants:**

This single-center, open-label, equivalence randomized clinical trial was conducted from January 1, 2016, to December 30, 2017, in Huashan Hospital, Fudan University. Adult patients with newly diagnosed IDA were enrolled. Participants were randomly assigned (1:1) to the oral iron supplements plus vitamin C group or the oral iron supplements–only group. Data analysis was performed from March to December 2018.

**Interventions:**

Patients were randomized to receive a 100-mg oral iron tablet plus 200 mg of vitamin C or a 100-mg iron tablet alone every 8 hours daily for 3 months.

**Main Outcomes and Measures:**

The primary outcome was the change in hemoglobin level from baseline to 2 weeks of treatment, and an equivalence margin of 1 g/dL in hemoglobin was chosen for the demonstration of comparable efficacy. Secondary outcomes included the change in the reticulocyte percentage after 2 weeks of treatment, the increase in hemoglobin level after 4 weeks of treatment, the increase in serum ferritin level after 8 weeks of treatment, and adverse events.

**Results:**

Among the 440 randomized patients (220 each in the oral iron supplements plus vitamin C group and iron-only group; 426 women [96.8%]; mean [SD] age, 38.3 [11.7] years), all were assessed for the primary outcome, and 432 (98.2%) completed the trial. From baseline to the 2-week follow-up, the mean (SD) change in hemoglobin level was 2.00 (1.08) g/dL in the oral iron supplements plus vitamin C group and 1.84 (0.97) g/dL in the oral iron supplements–only group (between-group difference, 0.16 g/dL; 95% CI, −0.03 to 0.35 g/dL), thus meeting the criteria for equivalence. The mean (SD) change in serum ferritin level from baseline to 8-week follow-up was 35.75 (11.52) ng/mL in the vitamin C plus iron group and 34.48 (9.50) ng/mL in the iron-only group (between-group difference, 1.27 ng/mL; 95% CI, −0.70 to 3.24 ng/mL; *P* = .21). There were no significant differences between the 2 groups regarding the rates of adverse events (46 [20.9%] vs 45 [20.5%]; difference, 0.4%; 95% CI, −6.7% to 8.5%; *P* = .82). No patient withdrew because of adverse events.

**Conclusions and Relevance:**

Among patients with IDA, oral iron supplements alone were equivalent to oral iron supplements plus vitamin C in improving hemoglobin recovery and iron absorption. These findings suggest that on-demand vitamin C supplements are not essential to take along with oral iron supplements for patients with IDA.

**Trial Registration:**

ClinicalTrials.gov Identifier: NCT02631668

## Key Points

**Question:**

Are the effects of oral iron supplements alone equivalent to a regimen of oral iron supplements plus vitamin C in the treatment of iron deficiency anemia?

**Findings:**

In this randomized clinical trial that included 440 adults with iron deficiency anemia, the mean change in hemoglobin level after 2 weeks was 2.00 g/dL in the oral iron supplements plus vitamin C group, compared with 1.84 g/dL in the oral iron supplements–only group. This difference met prespecified criteria for equivalence.

**Meaning:**

The use of oral iron supplements alone is comparable to a regimen of vitamin C supplemented with oral iron for patients with iron deficiency anemia.

## Teaser

This randomized clinical trial compares the equivalence and assesses the safety of oral iron supplements plus vitamin C vs oral iron supplements alone among patients with iron deficiency anemia.

## Introduction

Iron deficiency anemia (IDA) is associated with a decrease in erythropoiesis caused by a deficit in total body iron.^1^ Iron deficiency is the leading cause of anemia worldwide. According to the World Health Organization guideline,^2^ IDA affects 30% of the world’s population, indicating that it is a problem requiring attention.

Iron deficiency can be divided into 3 stages: prelatent iron deficiency, latent iron deficiency (also called iron-deficient erythropoiesis), and iron deficiency anemia (IDA).^2,3^ At the first stage, iron intake lower than the required amount causes progressive depletion of iron storage primarily in the liver and muscle cells. Patients at this stage generally have no symptoms, and the diagnosis of iron deficiency is made when levels of serum ferritin (the storage form of iron) decrease below 20 ng/mL (to convert to micrograms per liter, multiply by 1.0). Sustained iron storage depletion leads to the second stage of iron deficiency, iron-deficient erythropoiesis, in which iron deficiencies progress and begin to affect erythropoiesis. Despite an increased transferrin level, serum iron level decreases along with transferrin saturation. Erythropoiesis impairment appears when the serum iron level decreases to less than 50.3 μg/dL (to convert to micromoles per liter, multiply by 0.179) and transferrin saturation is less than 16%.^4^ Hemoglobin level is still within the normal range until the development of the IDA stage. Iron storage levels deplete to the point that they can no longer support the hemoglobin production and generate enough red blood cells (RBCs). Iron deficiency impairs RBC synthesis and hemoglobin production, leading to anemia.^5^

During the prelatent iron deficiency phase, an iron-rich diet can treat most cases. However, patients with IDA require iron supplements to replenish storage iron, restore normal hematopoiesis, treat anemia, and relieve symptoms.^3^

Oral iron supplementation is the primary approach to restore iron levels for patients with IDA. Numerous nonheme iron supplements are available, and ferrous sulfate and ferric succinate supplements are the most commonly used. Vitamin C is the only dietary constituent other than animal tissue that has been shown to promote iron absorption.^6,7,8,9^ Iron absorption occurs predominantly in the duodenum and upper jejunum, where ferrous iron can be transported into small intestine mucosal epithelial cells. When taken orally, iron is always oxidized to the Fe^3+^ state from its original form. It requires an acidic gastrointestinal environment to be dissolved adequately for absorption. Vitamin C can create a more acidic environment in the stomach and prevent the oxidization of ferrous iron to ferric iron.^10^ However, in a series of 12 individuals treated with iron during intake of a regular or vitamin C–supplemented diet,^8^ the effect of vitamin C on promoting iron absorption from a complete diet was far less pronounced than that from a single meal. The facilitating impact of vitamin C with food on iron status is minimal.^8,11^ Therefore, whether vitamin C has additional advantages, such as improving the efficacy of iron tablets and, thus, speeding up the recovery of anemia, remains poorly understood. Whether iron tablets with vitamin C supplements should be recommended is controversial.

To our knowledge, until now there has not been a randomized clinical trial (RCT) to assess whether vitamin C supplements are necessary for patients with IDA taking iron tablets. Therefore, it is necessary to conduct a rigorous RCT to assess the efficacy and safety of oral iron supplements alone or combined with vitamin C in patients with IDA. We designed a single-center, equivalence RCT to evaluate whether oral iron supplements alone were comparable with oral iron supplements plus vitamin C and to verify whether vitamin C routinely used with iron supplements can improve iron absorption.

## Methods

### Study Design

This 2-year, open-label, single-center, RCT was conducted at Huashan Hospital, Fudan University, Shanghai, China. All patients provided written informed consent before the commencement of the study. The institutional review board of Huashan Hospital, Fudan University, approved this research. This study follows the Consolidated Standards of Reporting Trials (CONSORT) reporting guideline.

**Figure 1.**
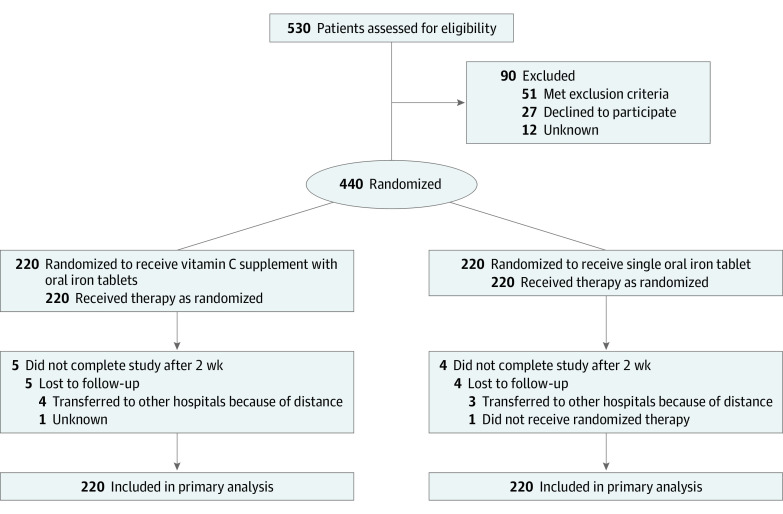
CONSORT Flow Diagram

Randomization of participants at a 1:1 ratio was performed using Stata statistical software version 11.0 (StataCorp) by a randomization procedure (Figure 1). Sequentially numbered, opaque, sealed envelopes ensured the concealment of randomization. The serial numbers on the outside of the envelopes were consistent with the patients’ visit numbers. After baseline measurements, the physician provided the sealed envelope marked with the eligible patient’s consulting number. Then the patient opened the sealed envelope, which contained the information on the assigned randomization group.

The intervention group received a 100-mg oral iron tablet (ferrous succinate, 100 mg/tablet) plus 200 mg of vitamin C (vitamin C, 100 mg/tablet) every 8 hours daily; the control group received a 100-mg iron tablet (ferrous succinate, 100 mg/tablet) every 8 hours daily. All patients took the supplements with warm water half an hour after a meal. Compliance was addressed by determining the actual number of iron and vitamin C tablets returned by the participants. The complete trial protocol can be seen in Supplement 1.

### Study Population

We screened patients newly diagnosed with IDA who had not received any iron supplement therapy in Huashan Hospital, Fudan University, from January 1, 2016, to December 30, 2017. Some of these patients were found to have anemia during a routine checkup. Most patients presented because of symptoms such as dizziness and palpitation. The nurse prechecked first. If the patients were found to have anemia, the nurse would recommend them to the anemia clinic to receive examination and treatment. Then our team screened these patients for enrollment.

The inclusion criteria were age 18 years or older, voluntarily signing the informed consent form, and meeting the diagnostic criteria for IDA, which include a hemoglobin level less than 13 g/dL for men or less than 12 g/dL for women (to convert hemoglobin to grams per liter, multiply by 10.0), mean corpuscular volume (MCV) less than 80 μm^3^ (to convert MCV to femtoliters, multiply by 1.0), mean corpuscular hemoglobin (MCH) less than 27 pg/cell, mean corpuscular hemoglobin concentration (MCHC) less than 32 g/dL (to convert MCHC to grams per liter, multiply by 10), serum ferritin level less than 14 ng/mL for women or 30 ng/mL for men, serum iron less than 39 μg/dL for women or 56 μg/dL for men, transferring saturation less than 20%, and total iron-binding capacity (TIBC) exceeding 428 μg/dL (to convert TIBC to micromoles per liter, multiply by 0.179). Exclusion criteria were pregnancy, severe uncorrectable bleeding, identified stomachache or intestinal ulcers, any inflammatory diseases, or identified gastrointestinal tumors.

### Study Monitor

Patients were treated for 3 months and assessed with a complete blood count every 2 weeks for 2 months; iron metabolism was measured at week 8. The primary outcome was the change in hemoglobin level from baseline to the 2-week follow-up. The secondary outcomes included the change in the reticulocyte percentage after 2 weeks of treatment, the increase in hemoglobin after 4 weeks of treatment, the increase in serum ferritin after 8 weeks of treatment, and adverse events. Exploratory outcomes included MCV, MCH, and MCHC levels every 2 weeks at all time points and serum iron level, transferring saturation, and TIBC at 8 weeks. The Department of Laboratory Medicine in Huashan Hospital, Fudan University, conducted the measurements and biochemical analyses.

### Statistical Analysis

Sample size calculations were performed for the change in hemoglobin level from baseline using an equivalence design; with bounds of 1 g/dL for the mean difference and a significance level of .05, a total sample size of 392 participants (assuming no difference between groups), a common SD of 1.5 g/dL, an allowable error of 0.5 g/dL, and an allocation ratio of 1:1 would correspond to a power of 90%. Considering a dropout rate of 10%, 440 patients in total were enrolled in the study.

Results with normal distributions were confirmed by a normal distribution test and were presented as mean (SD) values. Comparison of ages, the baseline of complete blood count, and iron metabolism parameters between the 2 groups was performed with the *t* test. Comparison of sexes and the incidence of adverse reactions between the 2 groups were based on 2-sided Pearson χ^2^ test. The 95% CIs for the difference of the changes of hemoglobin level between the 2 groups were calculated at each time point, and the equivalence was evaluated using the predefined margins of equivalence (±1 g/dL). Efficacy variables were analyzed on an intention-to-treat basis. If patients dropped out, missing data were imputed by the last observation carried forward method. For analysis of adverse events, patients who received at least 1 dose of study drug were included in the safety population. The threshold of a statistical significance was set as a *P* < .05. All tests were performed using Stata statistical software version 11.0 (StataCorp) from March to December 2018. The full statistical analysis plan is available in Supplement 2.

## Results

### Participants

Of 530 patients assessed for eligibility, 90 were excluded. The remaining 440 patients (mean [SD] age, 38.3 [11.7] years) underwent randomization. All patients completed the follow-up until 5 patients in the vitamin C plus iron group and 4 patients in the iron-only group dropped out after 2 weeks. The proportion of compliance was 98.2% (432 participants). Figure 1 shows the flow of participants in this trial.

Of the 440 patients, 426 (96.8%) were women with a mean age of 38.1 years (range, 18-90 years). The most common cause of IDA in women was menorrhagia due to uterine fibroids or endometriosis, which was found in 389 women (91.3%) in this study. Other reasons included hemorrhoidal hemorrhage (9 patients [2.1%]), vegetarian diet (7 patients [1.6%]), fecal occult blood positive due to gastrointestinal bleeding (4 patients [0.9%]), repeated hematuria (1 patients [0.2%]), and unknown (16 patients [3.8%]). Fourteen of the patients (3.2%) were men with a mean age of 61.4 years (range, 24-80 years), with causes of IDA including hemorrhoidal hemorrhage (5 patients [35.7%]), bleeding ulcer (4 patients [28.6%]), gastric cancer after surgery (3 patients [21.4%]), intestinal inflammation (1 patient [7.1%]), and colon cancer after surgery (1 patient [7.1%]). There were no significant differences between the 2 groups in terms of baseline characteristics (Table 1).

**Table 1.** Baseline Characteristics of Patients by Treatment Group

Characteristic	Mean (SD)	
	Vitamin C plus iron (n = 220)	Iron only (n = 220)
Sex, patients, No. (%)		
Female	215 (97.7)	211 (95.9)
Male	9 (2.3)	5 (4.1)
Age, y	38.0 (11.0)	39.6 (12.3)
Severity of anemia, patients, No.^a^		
Mild	105 (47.7)	109 (49.5)
Moderate	96 (43.6)	91 (41.4)
Severe	19 (8.6)	20 (9.1)
Hemoglobin, g/dL	8.76 (1.70)	8.82 (1.72)
Red blood cell count, ×10^6^/μL	4.18 (0.58)	4.17 (0.63)
Mean corpuscular volume, μm^3^	72.85 (9.29)	72.99 (9.00)
Mean corpuscular hemoglobin, pg/cell	21.51 (7.71)	22.38 (6.41)
Mean corpuscular hemoglobin concentration, g/dL	28.72 (1.94)	28.85 (3.19)
Red blood cell distribution width coefficient of variation, %	17.82 (2.65)	17.99 (3.16)
Reticulocyte, %	1.36 (0.87)	1.45 (0.94)
White blood cell count, /μL	6150 (3900)	5950 (2120)
Platelet count, ×10^3^/μL	314.02 (101.92)	304.52 (99.38)
Serum ferritin, ng/mL	5.89 (4.01)	6.34 (3.69)
Serum iron, μg/dL	23.12 (17.77)	25.08 (13.46)
Iron saturation, %	5.99 (1.97)	6.15 (3.69)
Total iron-binding capacity, μg/dL	431.01 (50.22)	423.46 (56.37)

SI conversion factors: To convert hemoglobin to grams per liter, multiply by 10.0; mean corpuscular volume to femtoliters, multiply by 1.0; mean corpuscular hemoglobin concentration to grams per liter, multiply by 10.0; platelet count to ×10^9^ per liter, multiply by 1.0; serum ferritin to micrograms per liter, multiply by 1.0; serum iron to micromoles per liter, multiply by 0.179; red blood cell count to ×10^12^ per liter, multiply by 1; total iron-binding capacity to micromoles per liter, multiply by 0.179; white blood cell count to ×10^9^ per liter, multiply by 0.001.

^a^ Severe anemia is defined as hemoglobin less than or equal to 6 g/dL, moderate anemia is defined as hemoglobin 6 to 9 g/dL, and mild anemia is defined as hemoglobin greater than 9 g/dL.

### Outcomes

#### Primary Outcome: Change in Hemoglobin Level

**Figure 2.**
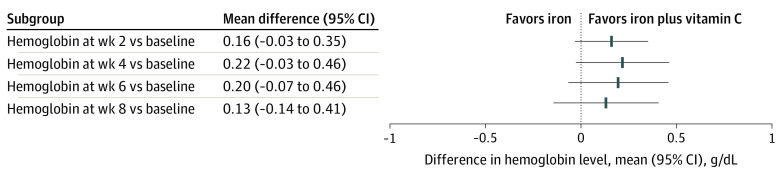
Differences of Changes in Hemoglobin Level Between the Vitamin C Plus Iron Group and the Iron-only Group The prespecified equivalence margin for the clinical significance of changes in hemoglobin between groups was 1 g/dL. Error bars indicate 95% CIs.

From baseline to 2-week follow-up, the mean (SD) change in hemoglobin level was 2.00 (1.08) g/dL in the vitamin C plus iron group and 1.84 (0.97) g/dL in the iron-only group (mean between-group difference, 0.16 g/dL; 95% CI, −0.03 to 0.35 g/dL). For the primary outcome, the change in hemoglobin level from baseline to 2-week follow-up was contained within the equivalence margin of 1 g/dL (Figure 2); thus, there was no significant difference in hemoglobin level increase between the 2 groups. Similarly, there was no significant difference in the change in hemoglobin level at weeks 4, 6, and 8 (Table 2).

**Table 2.** Changes in Red Blood Cell Parameters From Baseline to Weeks 2, 4, 6, and 8 and Iron Metabolism Parameters from Baseline to Week 8

Variable	Mean (SD)		Between-group difference (95% CI)
	Vitamin C plus iron	Iron only	
Change in hemoglobin level, g/dL			
2 wk	2.00 (1.08)	1.84 (0.97)	0.16 (−0.03 to 0.35)
4 wk	3.20 (1.32)	2.98 (1.28)	0.22 (−0.03 to 0.46)
6 wk	3.82 (1.37)	3.62 (1.45)	0.20 (−0.07 to 0.46)
8 wk	4.20 (1.47)	4.07 (1.48)	0.13 (−0.14 to 0.41)
Change in reticulocyte percentages			
2 wk	1.10 (1.18)	0.99 (1.06)	0.11 (−0.10 to 0.32)
4 wk	0.51 (1.05)	0.40 (1.29)	0.11 (−0.11 to 0.33)
6 wk	0.31 (0.88)	0.16 (0.86)	0.15 (−0.01 to 0.31)
8 wk	0.16 (0.82)	0.02 (0.81)	0.14 (−0.01 to 0.29)
Change in mean corpuscular volume, μm^3^			
2 wk	7.42 (4.67)	6.14 (4.06)	1.28 (0.46 to 2.09)
4 wk	10.52 (5.36)	9.25 (5.17)	1.27 (0.28 to 2.25)
6 wk	12.80 (5.89)	11.49 (5.84)	1.31 (0.21 to 2.41)
8 wk	14.16 (6.35)	12.72 (6.12)	1.44 (0.27 to 2.60)
Change in mean corpuscular hemoglobin, pg/cell			
2 wk	2.83 (1.77)	2.36 (1.49)	0.47 (0.16 to 0.77)
4 wk	4.87 (2.04)	4.29 (2.28)	0.58 (0.17 to 0.98)
6 wk	6.19 (2.40)	5.69 (2.61)	0.50 (0.03 to 0.97)
8 wk	7.18 (2.70)	6.47 (2.66)	0.71 (0.21 to 1.21)
Change in mean corpuscular hemoglobin concentration, g/dL			
2 wk	0.92 (1.02)	0.82 (0.79)	0.10 (−0.07 to 0.27)
4 wk	2.21 (1.23)	2.04 (1.22)	0.17 (−0.06 to 0.40)
6 weeks	3.10 (1.33)	2.82 (1.41)	0.28 (0.02 to 0.53)
8 weeks	3.47 (1.44)	3.27 (1.47)	0.20 (−0.07 to 0.47)
Change in red blood cell distribution width coefficient of variation, %			
2 wk	4.44 (2.49)	4.53 (2.41)	−0.09 (−0.55 to 0.37)
4 wk	3.61 (2.72)	3.49 (3.01)	0.12 (−0.42 to 0.66)
6 wk	2.54 (2.75)	2.22 (3.54)	0.32 (−0.27 to 0.91)
8 wk	−0.93 (2.95)	−0.53 (3.51)	−0.38 (−0.98 to 0.26)
Changes in iron metabolism parameters at wk 8			
Serum ferritin, ng/mL	35.75 (11.52)	34.48 (9.50)	1.27 (−0.70 to 3.24)
Serum iron, μg/dL	70.11 (30.67)	73.41 (25.47)	−3.30 (−8.55 to 1.96)
Transferrin saturation, %	24.25 (8.84)	27.11 (9.86)	−2.86 (−4.61 to −1.11)
Total iron-binding capacity, μg/dL	−122.63 (27.77)	−112.79 (33.80)	−9.83 (−15.59 to −4.08)

SI conversion factors: To convert hemoglobin to g/L, multiply by 10.0; mean corpuscular volume to fL, multiply by 1.0; mean corpuscular hemoglobin concentration to g/L, multiply by 10; serum ferritin to μg/L, multiply by 1.0; serum iron to μmol/L, multiply by 0.179; total iron-binding capacity to μmol/L, multiply by 0.179.

#### Secondary Outcomes: Changes in RBC Parameters

The median (interquartile range) change in reticulocyte percentage after a 2-week follow-up was 1.03% (0.74%-1.20%) in the vitamin C plus iron group and 1.04% (0.50%-1.20%) in the iron-only group (between-group difference, 0.11%; 95% CI, 0.10% to 0.32%); the difference was not significant. Similar results were obtained at week 4, 6, and 8 showing no significant differences between the 2 groups (Table 2).

MCV and MCH levels increased slightly (all 95% CIs were positive) in the vitamin C plus iron group at all follow-up points (Table 2). The increase in MCHC level was higher in the vitamin C plus iron group at 6 weeks, with mean (SD) changes in MCHC of 3.10 (1.33) g/dL in vitamin C plus iron group and 2.82 (1.41) g/dL in the iron-only group (between-group difference, 0.28 g/dL; 95% CI, 0.02-0.53 g/dL). Changes of RBC distribution width coefficient of variation showed no significant difference between the 2 groups at all follow-up points (Table 2).

### Iron Metabolism Parameters

Likewise, no difference was observed between groups for the change in serum ferritin level from baseline to 8-week follow-up. The mean (SD) change was 35.75 (11.52) ng/mL in the vitamin C plus iron group and 34.48 (9.50) ng/mL in the iron-only group (mean between-group difference, 1.27 ng/mL; 95% CI, −0.70 to 3.24 ng/mL; *P* = .21). The mean changes in serum iron, transferring saturation, and TIBC were also comparable between the 2 groups (Table 2).

### Adverse Events

The treatment was similarly well tolerated during 8 weeks of follow-up. The most frequent adverse events were stomach upset, nausea, and acid reflux (Table 3). The proportion of patients with adverse events was comparable between the 2 groups, 46 (20.9%) in the vitamin C plus iron group and 45 (20.5%) in the iron-only group after 2 weeks of follow-up (difference, 0.4%; 95% CI, −6.7% to 8.5%; *P* = .82). After 2 weeks of treatment, 68 patients with adverse events felt better and another 23 patients could tolerate the adverse events. No patients opted out because of adverse events (Table 3).

**Table 3.** Adverse Events by Treatment Group

Adverse event	Patients, No. (%)	
	Vitamin C plus iron (n = 220)	Iron only (n = 220)
Stomach upset, nausea and acid reflux	30 (13.64)	29 (13.18)
Constipation	7 (3.18)	8 (3.64)
Diarrhea	4 (1.82)	3 (1.36)
Xerostomia	3 (1.36)	2 (0.91)
Abdominal pain	2 (0.91)	2 (0.91)
Chest distress	0	1 (0.45)

## Discussion

These findings demonstrate that oral iron supplements alone provide hemoglobin level and iron storage recovery efficacy equivalent to that of oral iron supplemented with vitamin C in patients with IDA. To our knowledge, this RCT is the first to evaluate the efficacy and safety of vitamin C supplements combined with oral iron in patients with IDA.

Oral iron provides an inexpensive and effective means of restoring iron balance in patients with IDA. Vitamin C is the only dietary constituent other than animal tissue that has been shown to promote the absorption of nonheme iron in humans.^9^ Thus, some clinicians, who believe that vitamin C can improve the efficacy of oral iron and speed up the treatment of anemia, recommend taking vitamin C supplements combined with oral iron tablets. However, the absolute dose of vitamin C did not appear to be associated with iron absorption with a complete diet, and the improvement in iron status was not noticeable,^8,11^ which was consistent with our findings that oral iron alone provides efficacy equivalent to that of oral iron with vitamin C. The changes in hemoglobin level were similar between the 2 groups at weeks 2, 4, 6, and 8. The mean difference in hemoglobin level change was within the equivalence margin of ±1 g/dL, which is the threshold for clinically significant change recommended by hematologists. Notably, the changes in MCV from baseline to all time points were slightly greater in the vitamin C plus iron group. MCV is a measure of the mean size of RBCs, and patients with IDA typically have lower than normal values. The RBC parameters MCH, MCV, and MCHC are more sensitive and helpful to monitor the response to a treatment.^12^ However, the aim of treatment was restoring hemoglobin levels and replenishing iron stores, neither of which was sensitive to the treatment. Iron metabolism parameters such as serum ferritin, serum iron, iron saturation, and TIBC at week 8 were comparable between the 2 groups. Adverse events were also equivalent between the 2 groups.

These results challenge the recommendation to take vitamin C supplements with oral iron to improve the efficacy and speed up the recovery from anemia. Iron is absorbed as the ferrous salt (Fe^++^) in a mildly acidic medium. In theory, vitamin C is helpful in its absorption. However, in light of the results of this RCT, vitamin C may be less beneficial than reported and expected.

### Limitations

This study has some limitations. First, 426 of the included patients were women and 19 were men, which was an imbalance. Second, the overall follow-up period was not long enough. We found that iron deficiency anemia relapsed easily if the underlying causes, such as iron-absorption defects and bleeding, were not investigated and addressed. Some patients came back to our clinic approximately 1 year after the trial ended because of relapse. The time when iron deficiency reappeared again and whether the proportion of relapse was comparable for the 2 groups were both unknown. Future research should extend the observation period to obtain more accurate and reliable results.

## Conclusions

The findings of this equivalence RCT demonstrate that in patients with IDA, taking oral iron alone was equivalent to taking oral iron supplemented with vitamin C in improving hemoglobin level and iron stores. Our results suggest that vitamin C is not essential for patients with IDA.

## Notes

Supplement 1. **Trial Protocol**

Supplement 2. **Statistical Analysis Plan**

Supplement 3. **Data Sharing Statement**
