# Supplementary material for: The Efficacy and Safety of Vitamin C for Iron Supplementation in Adult Patients With Iron Deficiency Anemia: A Randomized Clinical Trial
Source: JAMA Netw Open. 2020 Nov 2;3(11):e2023644. doi: 10.1001/jamanetworkopen.2020.23644 (PMC7607440; doi:10.1001/jamanetworkopen.2020.23644)
Supplement: Supplement 1. — Trial Protocol [file jamanetwopen-e2023644-s001.pdf]

The Efficacy and Safety of Vitamin C for Iron Supplementation in Adult patients with Iron Deficiency Anemia: A Randomized Clinical Trial

Registry: Clinicaltrials.gov (NCT02631668)

Effective date: 1/2016 - 12/2018

Research Unit: Department of hematology, Huashan Hospital

Scientific Participants:

Xiaoqin Wang (Primary Investigator), MD, PhD,

Deputy Director of Department of hematology, Huashan Hospital, Fudan University

Email: wangxiaoqin@shmu.edu.cn

Nianyi Li, MD,PhD

Department of Hematology, Huashan Hospital, Fudan University

Email: nianyili@gmail.com

Wei Wang, MD

Department of Hematology, Huashan Hospital, Fudan University

Email: ciwei108@sina.com

Guangjie Zhao, MD

Department of Hematology, Huashan Hospital, Fudan University

Email: zhaoguangjie@163.com

Contents

1. Background

2. Objective

3. Research Design

4. Inclusion and Exclusion

5. Medication Regimen

6. Randomization

7. Sample Size

8. Endpoint Evaluation

9. Study Flow Chat

10. Regulations of Reporting of Adverse Events

11. Data Management

12. Statistical Analysis

13. Quality Control

14. Ethical standard

15. References

16. Report Form of Adverse Events

1. Background

Iron deficiency anemia (IDA) is associated with a decrease in erythropoiesis caused by a deficit in total body iron.1 Iron deficiency is the leading cause of anemia worldwide. According to the WHO Guideline, IDA affects 30% of the world's population, indicating that it is still a problem requiring attention.2

Iron deficiency can be divided into three stages: pre-latent iron deficiency, latent iron deficiency (also called iron-deficient erythropoiesis, IDE), and iron deficiency anemia (IDA). At the first stage, a lower iron intake (than the requirement) causes progressive depletion of iron storage primarily in the liver and muscle cells. This stage generally has no symptoms and is often diagnosed when serum ferritin (storage form of iron) levels drop below 20 ug/L. Continued iron storage depletion leads to the second stage, IDE phase, when iron deficiencies progress and begin to affect Erythropoiesis. In spite of an increased transferrin level, serum iron level decreases along with transferrin saturation. Erythropoiesis impairment begins when the serum iron level falls to less than 9 umol/L and transferrin saturation is less than 16%.3 Hemoglobin levels are still within the normal range until IDA stage. Iron storages are depleted to a point where it can no longer support the hemoglobin production required to make enough red blood cells. Deficiency impairs RBC synthesis, and hemoglobin production declines to the point where anemia develops.4

During the pre-latent iron deficiency phase, most situations can be treated through an iron-rich diet. However, patients diagnosed with IDA need iron supplements promptly to restore the symptoms, and more importantly, investigating and addressing the underlying causes of IDA such as iron-absorption defects and bleeding.

Oral iron supplementation is the major way to restore iron levels for IDA patients. Numerous non-heme iron supplements are available, among which the most commonly used are ferrous sulfate and ferric succinate. Vitamin C is the only dietary constituent other than animal tissue that has been shown to promote iron absorption.5-8 Iron absorption occurs predominantly in the duodenum and

upper jejunum, where only ferrous iron can be transported into small intestine mucosal epithelial cells. When taken orally, iron is always oxidized to the Fe3+ state from different original forms. It requires an acidic GI environment to properly dissolve and to be available for absorption. Vitamin C can create a more acidic environment in the stomach and prevent the oxidization of ferrous iron to ferric iron.9 However, in a series of 12 individuals treated with iron during an intake of a normal or vitamin C supplemented diet, the augmentation of vitamin C on iron absorption from a complete diet is far less pronounced than that from a single meal. The facilitating effect in iron status of vitamin C with a complete diet has been minimal.7,10 Therefore, whether Vitamin C has additional advantages, such as improving the efficacy of the iron tablets, speeding up the recovery of anemia, should be reconsidered. Some doctors might recommend taking iron tablets with vitamin C supplements while others may not. Their experience-based recommendations lack a support from clinical evidence through Randomized Controlled Trial (RCT), which is considered to be the most reliable form of scientific evidence.

With the rising of concept of evidence-based medicine, some shortcomings of empirical medicine were gradually exposed. For example, most experts used lidocaine as standard treatment for acute myocardial infarction until 1999, when Sadowski et al evaluated totally 21 randomized controlled trials (RCTs). The result showed that in patients receiving lidocaine treatment, although the incidence of ventricular fibrillation in patients with acute myocardial infarction was reduced, the mortality increased significantly. It concluded that using lidocaine to prevent ventricular fibrillation in patients with acute myocardial infarction was not recommended11. We believe that pathophysiological or mechanistic feasibility may not be able to achieve desired results in patients. Evidence-based medicine emphasizes that randomized controlled trail (RCT), as a gold standard for efficacy evaluation, should be apply for all therapies.

Therefore, it is necessary to conduct a rigorous RCT study to evaluate whether vitamin C can increase the iron supplements efficacy. We designed a single-center,

randomized controlled, equivalent clinical trial to study the efficacy and safety between taking iron pills only and with vitamin C supplements. The aim of this study was to evaluate whether vitamin C supplements can speed up the recovery of IDA and whether this produces any side effects, such as increased gastrointestinal irritation stimulated by iron therapy itself.

2. Objectives

2.1 Primary Objective

To explore whether oral iron tablets supplemented with vitamin C can speed up the recovery of IDA stimulated by iron therapy itself.

2.2 Secondary Objective

To explore whether vitamin C supplement increases the incidence of the adverse events, such as increased gastrointestinal irritation stimulated by iron therapy itself.

3. Research Design

This study is an open-label, randomized, single-center clinical trial. Patients will be randomized to two groups using STATA 11.0 software. Participants are randomly assigned (1:1) to the oral iron tablets supplemented with vitamin C group or the oral iron tablets used as single-drug treatment group. Patients are randomized to receive 100mg oral iron tablet plus 200mg vitamin C supplements every 8 hours daily (n=220) or 100mg iron tablet every 8 hours (n=220) for 3 months.

4. Inclusion and Exclusion

4.1 Diagnostic Criteria for IDA

Hemoglobin level less than 130g/L for men or 120 g/L for women, Mean Corpuscular Volume (MCV) less than 80fl, Mean Corpuscular Hemoglobin (MCH)

less than 27pg, Mean Corpuscular Hemoglobin Concentration (MCHC) less than 320g/L, Serum Ferritin level less than 14ug/L for women or 30ug/L for men, Serum iron less than 7umol/L for women or 10um/L for men, Transferring saturation (TFs) less than 0.20, Total Iron Bind Capacity (TIBC) exceeding 76.6umol/L

4.2 Inclusion Criteria

Adult outpatients, who were newly diagnosed with IDA from 1/1/2016 to 12/30/2017 in Huashan Hospital, Fudan University, and had not received any iron-supplement therapy, were screened for enrollment. The inclusion criteria were age of at least 18 years old, meeting IDA diagnostic criteria, and being voluntary to sign the informed consent.

4.3 Exclusion Criteria

(1) Pregnancy; (2) Severe stomachache or intestinal ulcers; (3) Intolerable an oral iron treatment; (4) Serious uncorrectable bleeding; (5) Drug Allergy; (6) Severe liver dysfunction (The levels of ALT and AST increase to two times higher than normal); (7) Cardiovascular Disease; (8) Renal insufficiency; (9) Participate in other clinical trials; (10) Disagree to sign the informed consent.

4.4 Termination Criteria

(1) Subjects with poor compliance or special reasons, and research drug cannot be used on time; (2) Patients use other drugs that may affect efficacy of research drug or tolerance judgment; (3) Subjects are reluctant to continue clinical trials,; (4) Serious adverse effects occur in clinical trial; (5) Other symptoms of concurrent disease worsened in clinical trial and need urgent treatment.

5. Medication Regimen

5.1 Research drug.

Drug	Form	Specification	Storage	Lot.	Company
ferrous succinate	tablet	0.1g/tablet	Shading, Airtight，Room temperature	SFDA number, H20083003	Sichuan, Aobang pharmaceutical co., Ltd.
Vitamin C	tablet	0.1g/tablet	Shading, Airtight，Room temperature	SFDA number, H42020614	Hubei, Huazhong, pharmaceutical co., Ltd.

5.2 Treatment Regimen

Code	Group	Treatment
A	Intervention group	Ferrous succinate, 100mg, tid. Vitamin C, 200mg tid.
B	Control group	Ferrous succinate, 100mg, tid.

5.3 Dosing Compliance

The box and aluminum cardboard of medication packages need return to investigators when follow up. Patients should be emphasized to return the entire pharmaceutical package, including the one remaining pharmaceutical package, which can help researchers to calculate the information of dosing. Patients who took vitamin C also needed to return drug bottle every two weeks, which can help us to determine how much drug was available. Dosing compliance is defined as the ratio of actual dosage to the standard dosage. Actual dosage must within ±20% of

the standard. Chi-square test will be performed to compare the differences in compliance between two groups.

5.4 Drug Storage

The research drugs are stored by research unit, distributed to the patients in batches, and stored in a dry, dark environment.

6.Randomization

Randomization is performed using the statistic software STATA 11.0 by a centralized randomization procedure. The opaque, sealed and sequentially numbered randomization envelopes will be shuffled. After baseline measurements, eligible patients open a sealed envelope containing the information of the assigned randomization group at 1:1 ratio.

7. Sample Size

Sample size calculations were performed for change in Hb from baseline using an equivalence design, with bounds of ±10g/L for the mean difference, alpha=5%, beta=10%, and a significance level of 0.05, assuming no difference between groups and a common SD of 15g/L, allowable error of 5g/L; a total sample size of 392 participants assuming an allocation ratio of 1:1 would correspond to a power of 90%. Considering a dropout rate of 10%, 440 patients in total were enrolled in the study.

8. Endpoint evaluation

8.1 Primary endpoint

The primary endpoint is change in Hemoglobin (Hb) from baseline to 2 weeks of medication follow-up.

8.2 Secondary endpoints

- Change in the Reticulocyte percentage (Ret%) after 2 weeks of treatment.
- Increase in Hemoglobin after 4 weeks of medication follow-up.
- Increase in Serum Ferritin after 8 weeks of treatment.
- Adverse events.
- Exploratory outcomes include MCV, MCH, and MCHC every 2 weeks at all time points and serum iron, TFs, TIBC at 8 weeks.

9. Study Flow Chat

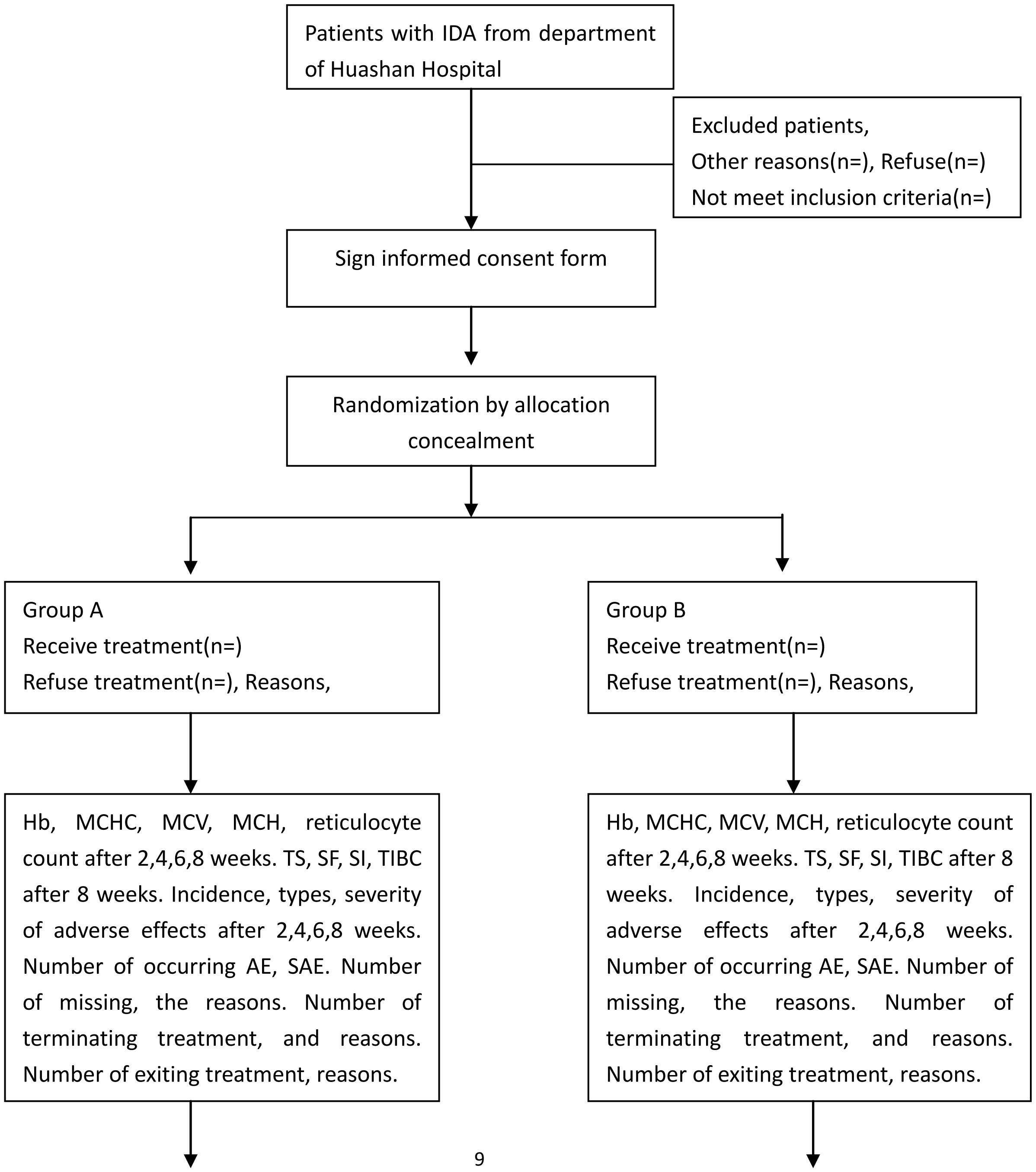

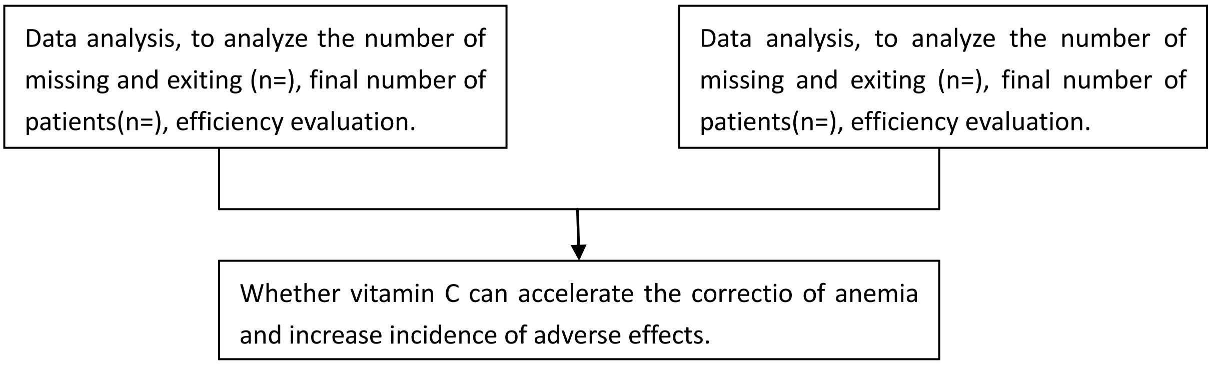

10. Regulations of Reporting of Adverse Events

10.1 Adverse events (AE)

Adverse events (AEs) refer to unfavorable medical events, which occur during clinical trials, whether the events are relevant to the trial or not. The investigators should follow up the adverse events until they disappear or stabilize and analysis the pathogens to determine the relationship between adverse event and research drug, which can be divided into: certainly related, possibly related, possibly unrelated, and certainly irrelevant. All adverse events that occur during the study must be reported in the adverse event table.

10.1.1 Classification of relation between adverse events and drugs

	Have Temporal relation with Research drug	Conform to the types of adverse effects	Adverse events alleviate after discontinuation	Adverse events present again after dosing	Situation can't be explained by progression and treatment
Certainly related	+	+	+	+	+
Very Possibly related	+	+	+	?	+
Possibly related	+	+	±	?	-
Possible unrelated	-	-	±	?	±

Certainly unrelated	-	-	-	-	-

Note：+, positive；- negative；± Uncertain；？ unknown

10.1.2 Judgment for severity of adverse events

Mild AE	Mild symptoms and do not develop, generally no treatment
Moderate AE	Obvious symptoms, Moderate damage to organs or system
Severe AE	Serious damage to organs or system, shorten or endanger life

10.2 Serious Adverse Event, SAE

SAE is adverse events that resulted in death, life-threatening situation, hospitalization, lengthening the time of hospitalization, disability, congenital abnormalities or birth defects. Patients are advised to return to the hospital in time or go to local hospital for treatment. The primary investigator or other participants should be present when patients occurred SAE. If necessary, the trail should be stopped immediately. Primary investigator should report the events to corresponding administrative department or the ethics committee of institution within 24 hours. The presentation of serious adverse events should use standard form provided by SFDA.

10.3 Pregnancy

The drugs in this study are safe for pregnant women. If patients are pregnant during the trial, the pregnancy report form of clinical trial is required. They need to be followed up to the birth of fetus. Investigators need close observation of the details of birth to know about the events of abortion, automatic termination of pregnancy and fetal malformation, etc.

10.4 Overdose

Overdose should be reported to clinical researcher within 24 hours. Researchers should report the corresponding medication. If it occurs serious consequences or symptoms that meet SAE assessment criteria, clinicians should take rescue measures and report to relevant administrative department of ethics committee within 24 hours. Primary investigator fills SAE report form.

11. Data management

The researchers must ensure the authenticity, completeness and reliability of clinical data. Any corrections should be underlined, indicated, signed and dated by the investigators. Original records should not be covered. Items for laboratory examination should be complete. Clinical data must be input by professional person. Dr. Wang will monitor multiple aspects of data validity, including consent documents, inclusion and exclusion criteria, AEs and other important daily data. The data will be kept for more than 5 years.

12. Statistical analysis.

Analysis of primary endpoint and secondary endpoint are following the basic principles of intention to Treat (ITT) approach to define the full analysis set. All randomized patients will be included in full analysis set for analysis. The missing data of efficacy evaluation will be estimated by last observation carried forward (LOCF).

Baseline characteristics and summary statistics will be described by treatment group. Continuous variables will be summarized using descriptive statics, such as numbers of subjects, means with standard deviations, medians with interquartile ranges for non-normal data, and categorical data by counts and proportions.

Qualitative variables will be summarized by frequency and percentage.

Results with normal distributions are presented as mean values (±SD).

Comparisons of ages, the baseline of Complete Blood Count and iron metabolism

parameters between the study groups are performed with a t-test. Comparisons of genders and the incidence of adverse reactions between the two groups, which do not subject to normal distributions, will base on a X2 test. Confidence intervals (95%) for the difference of the changes in Hb level between two groups were calculated at each time point, and the equivalence was evaluated using the predefined margins of equivalence (±10g/L). Efficacy variables were analyzed on an intention-to-treat. If patients drop out, missing data were imputed by Last-Observation-Carried-Forward (LOCF) method. For the analysis of adverse events, patients who accepted oral iron tablets were included in the safety population. The threshold of a statistical significance is set as a P-value smaller than 0.05. All tests were performed using STATA version 11.0.

13. Quality Control

(1) The research group should include one primary investigator and 2-4 research members. The study should be conducted strictly in the accordance to the research procedure.

(2) The research group must establish standard operating procedures and quality control procedures for clinical data, which should be measured in the units specified by state. Clinical reports should include dates, types of tests, results, normal ranges, and researchers' signatures.

(3) Ethics committee and primary investigator should review the implementation of clinical trials on time and check the subjects' compliance and data authenticity.

14. Ethical Standard

The ethics committee of Huashan Hospital, Fudan University approved the research plan, research form and informed consent form. Dr. Wang, the research director, is responsible for submitting the protocol, the informed consent and other relevant document to the ethical committee and obtaining approval for trial. The investigators or authorized person will be responsible for explaining the benefits and risks in this study to each patient. The patients should sign informed

consent before they are enrolled this study. All informed consent forms signed and dated by patients or investigators keep his/her legal representative. Any changes to the research protocol must be approved by the ethics committee. The clinical trial is followed up by Ethics Committee and in compliance with Helsinki Declaration.

14. References

1. Brugnara C. A hematologic "gold standard" for iron-deficient states? *Clin. Chem.* Jul 2002;48(7):981-982.

2. Camaschella C. Iron-deficiency anemia. *N. Engl. J. Med.* May 7 2015;372(19):1832-1843.

3. Moretti D, Goede JS, Zeder C, et al. Oral iron supplements increase hepcidin and decrease iron absorption from daily or twice-daily doses in iron-depleted young women. *Blood.* Oct 22 2015;126(17):1981-1989.

4. By Ronald Hoffman EJB, Jr., Leslie E. Silberstein, Helen Heslop, John Anastasi, Jeffrey Weitz. *Hematology: Basic Principles and Practice..*

: Elsevier Health Sciences; 2017.

5. Sayers MH, Lynch SR, Charlton RW, Bothwell TH, Walker RB, Mayet F. Iron absorption from rice meals cooked with fortified salt containing ferrous sulphate and ascorbic acid. *Br. J. Nutr.* May 1974;31(3):367-375.

6. Cook JD, Monsen ER. Vitamin C, the common cold, and iron absorption. *Am. J. Clin. Nutr.* Feb 1977;30(2):235-241.

7. Cook JD, Reddy MB. Effect of ascorbic acid intake on nonheme-iron absorption from a complete diet. *Am. J. Clin. Nutr.* Jan 2001;73(1):93-98.

8. Hallberg L, Brune M, Rossander L. Effect of ascorbic acid on iron absorption from different types of meals. Studies with ascorbic-acid-rich foods and synthetic ascorbic acid given in different amounts with different meals. *Hum. Nutr. Appl. Nutr.* Apr 1986;40(2):97-113.

9. Rocha DD, Capanema FD, Netto MP, de Almeida CAN, Franceschini SDC, Lamounier JA. Effectiveness of fortification of drinking water with iron and vitamin C in the reduction of anemia and improvement of nutritional status in children attending day-care centers in Belo Horizonte, Brazil. *Food and Nutrition Bulletin.* Dec 2011;32(4):340-346.

10. Hunt JR, Gallagher SK, Johnson LK. Effect of ascorbic acid on apparent iron absorption by women with low iron stores. *Am. J. Clin. Nutr.* Jun 1994;59(6):1381-1385.

11. Sadowski ZP, Alexander JH, Skrabucha B, et al. Multicenter randomized trial and a systematic overview of lidocaine in acute myocardial infarction. *Am. Heart J.* May 1999;137(5):792-798.

15. Serious Adverse Event Report Form

Project number/name			
Primary investigator		Tel	
Institution			
Sponsor		Tel	
**1.Information of subjects**<br>Code of subject<br>Time of happening SAE			
**2. Description of SAE (General conditions, examinations, cause analysis, and**			

treatment)

3. Information of research drug

Name of research drug (Chinese)

Name of research drug (English)

Dosage per day, Administration Route,

Date, Drug combination,

4. Serious adverse events

4.1. Classification of SAE

Hospitalization lengthening time of Hospitalization Disability

Influence the work congenital malformation life-threatening

Others

4.2 Relation between SAE and research drug

Certainly, related very possibly related possibly related

Possibly unrelated cannot evaluate

5. Management for research drug

Continue treatment reduce the dosage transient termination

terminate treatment

6. Transformation of SAE

Symptom vanish, no complication Symptom vanish, complications.

Persistent symptom, to date Under treatment

Death, Date of death

7. Concomitant medications and underlying diseases (list combined medications and underlying diseases, include the dosage, administration route and duration)

8. Whether adverse events are expected

8.1. SAE is an expected adverse event and has been indicated in research plan and informed consent form.

8.2. SAE is not an expected adverse event and has been indicated in research plan and informed consent form.

9. measures that have taken

Whether researchers modify research plan or informed consent form to avoid this risk.

No

Yes

Have modified research plan or informed consent form to avoid this risk.

Ready to modify research plan or informed consent form

Signature of Primary investigator： **Date**

Recommended form of reviewing

First review of ethics committee

Emergency meeting

Bulletin

Signature of Vice Chairman

Date

377
