# Supplementary material for: The Efficacy and Safety of Vitamin C for Iron Supplementation in Adult Patients With Iron Deficiency Anemia: A Randomized Clinical Trial
Source: JAMA Netw Open. 2020 Nov 2;3(11):e2023644. doi: 10.1001/jamanetworkopen.2020.23644 (PMC7607440; doi:10.1001/jamanetworkopen.2020.23644)
Supplement: Supplement 2. — Statistical Analysis Plan [file jamanetwopen-e2023644-s002.pdf]

The Efficacy and Safety of Vitamin C for Iron Supplementation in Adult Patients with Iron Deficiency Anemia

A single center, Randomized Control Study

Huashan Hospital authorization ID： KY2015-270

ClinicalTrials.gov ID: NCT02631668

Statistical Analysis Plan

Statistical Advisor:

Xiaoqin Wang, MD, Ph.D., Department of Hematology, Huashan Hospital, Fudan University

Statistical Analyst:

Nianyi Li, MD, Department of Hematology, Huashan Hospital, Fudan University

Abbreviations and Statistic descriptions

CRF	Case Report Form
FAS	Full Analysis Set
Q	Quartile
Max	Maximum
Min	Minimum
PPS	Per Protocol Set
SS	Safety Set
SD	Standard Deviation
CI	Confidence Interval
ITT	Intent to Treat
LOCF	Last Observation Carried Forward
AE	Adverse Event
SAE	Serious Adverse Event
MCV	Mean Corpuscular Volume
MCH	Mean Corpuscular Hemoglobin
MCHC	Mean Corpuscular Hemoglobin Concentration
Hb	Hemoglobin
SI	Serum Iron
TIBC	Total Iron Binding Capacity
SF	Serum Ferritin
TS	Transferrin Saturation
ID	Iron Deficiency
IDE	Iron Deficiency erythropoiesis
IDA	Iron Deficiency Anemia
RCT	Randomized Control Study

Background

Iron deficiency anemia (IDA) is associated with a decrease in erythropoiesis caused by a deficit in total body iron.[1] Iron deficiency is the leading cause of anemia worldwide. According to the WHO Guideline, IDA affects 30% of the world's population, indicating that it is still a problem requiring attention.[2]

Iron deficiency can be divided into three stages: pre-latent iron deficiency, latent iron deficiency (also called iron-deficient erythropoiesis, IDE), and iron deficiency anemia (IDA). At the first stage, a lower iron intake (than the requirement) causes progressive depletion of iron storage primarily in the liver and muscle cells. This stage generally has no symptoms and is often diagnosed when serum ferritin (storage form of iron) levels drop below 20 ug/L. Continued iron storage depletion leads to the second stage, IDE phase, when iron deficiencies progress and begin to affect Erythropoiesis. In spite of an increased transferrin level, serum iron level decreases along with transferrin saturation. Erythropoiesis impairment begins when the serum iron level falls to less than 9 umol/L and transferrin saturation is less than 16%.[3] Hemoglobin levels are still within the normal range until IDA stage. Iron storages are depleted to a point where it can no longer support the hemoglobin production required to make enough red blood cells. Deficiency impairs RBC synthesis, and hemoglobin production declines to the point where anemia develops.[4]

During the pre-latent iron deficiency phase, most situations can be treated through an iron-rich diet. However, patients diagnosed with IDA need iron supplements promptly to restore the symptoms, and more importantly, investigating and addressing the underlying causes of IDA such as iron-absorption defects and bleeding.

Oral iron supplementation is the major way to restore iron levels for IDA patients. Numerous non-heme iron supplements are available, among which the most commonly used are ferrous sulfate and ferric succinate. Vitamin C is the

only dietary constituent other than animal tissue that has been shown to promote iron absorption.[5-8] Iron absorption occurs predominantly in the duodenum and upper jejunum, where only ferrous iron can be transported into small intestine mucosal epithelial cells. When taken orally, iron is always oxidized to the Fe3+ state from different original forms. It requires an acidic GI environment to properly dissolve and to be available for absorption. Vitamin C can create a more acidic environment in the stomach and prevent the oxidization of ferrous iron to ferric iron.[9] However, in a series of 12 individuals treated with iron during an intake of a normal or vitamin C supplemented diet, the augmentation of vitamin C on iron absorption from a complete diet is far less pronounced than that from a single meal. The facilitating effect in iron status of vitamin C with a complete diet has been minimal.[7,10] Therefore, whether Vitamin C has additional advantages, such as improving the efficacy of the iron tablets, speeding up the recovery of anemia, should be reconsidered. Some doctors might recommend taking iron tablets with vitamin C supplements while others may not. Their experience-based recommendations lack a support from clinical evidence through Randomized Controlled Trial (RCT), which is considered to be the most reliable form of scientific evidence.

With the rising of concept of evidence-based medicine, some shortcomings of empirical medicine were gradually exposed. For example, most experts used lidocaine as standard treatment for acute myocardial infarction until 1999, when Sadowski et al evaluated totally 21 randomized controlled trials (RCTs). The result showed that in patients receiving lidocaine treatment, although the incidence of ventricular fibrillation in patients with acute myocardial infarction was reduced, the mortality increased significantly. It concluded that using lidocaine to prevent ventricular fibrillation in patients with acute myocardial infarction was not recommended[11]. We believe that pathophysiological or mechanistic feasibility may not be able to achieve desired results in patients. Evidence-based medicine emphasizes that randomized controlled trail (RCT), as a gold standard for efficacy evaluation, should be apply for all therapies.

Therefore, it is necessary to conduct a rigorous RCT study to evaluate whether vitamin C can increase the iron supplements efficacy. We designed a single-center, randomized controlled, equivalent clinical trial to study the efficacy and safety between taking iron pills only and with vitamin C supplements. The aim of this study was to evaluate whether vitamin C supplements can speed up the recovery of IDA and whether this produces any side effects, such as increased gastrointestinal irritation stimulated by iron therapy itself.

Primary Objective

To explore whether oral iron tablets supplemented with vitamin C can speed up the recovery of IDA stimulated by iron therapy itself.

Secondary Objective

To explore whether vitamin C supplement increases the incidence of the adverse events, such as increased gastrointestinal irritation stimulated by iron therapy itself.

Research Design

This study is an open-label, randomized, single-center clinical trial. Patients will be randomized to two groups using STATA 11.0 software. Participants are randomly assigned (1:1) to the oral iron tablets supplemented with vitamin C group or the oral iron tablets used as single-drug treatment group. Patients are randomized to receive 100mg oral iron tablet plus 200mg vitamin C supplements every 8 hours daily (n=220) or 100mg iron tablet every 8 hours (n=220) for 3 months.

Drugs

Research drugs

Drug	Form	Specification	Storage	Lot.	Company

ferrous succinate	tablet	0.1g/tablet	Shading, Airtight，Room temperature	SFDA number, H20083003	Sichuan, Aobang pharmaceutical co., Ltd.
Vitamin C	tablet	0.1g/tablet	Shading, Airtight，Room temperature	SFDA number, H42020614	Hubei, Huazhong, pharmaceutical co., Ltd.

The Vitamin C was provided by Hubei Huazhong Pharmaceutical Co., Ltd. in free of charge.

Treatment regimen

Code	Group	Treatment
A	Intervention group	Ferrous succinate, 100mg, tid. Vitamin C, 200mg tid.
B	Control group	Ferrous succinate, 100mg, tid.

Randomization

Randomization is performed using the statistic software STATA 11.0 by a centralized randomization procedure. The opaque, sealed and sequentially numbered randomization envelopes will be shuffled. After baseline measurements, eligible patients open a sealed envelope containing the information of the assigned randomization group at 1:1 ratio.

Sample Size

Sample size calculations were performed for change in Hb from baseline using an equivalence design, with bounds of ±10g/L for the mean difference, alpha=5%, beta=10%, and a significance level of 0.05, assuming no difference between

groups and a common SD of 15g/L, allowable error of 5g/L; a total sample size of 392 participants assuming an allocation ratio of 1:1 would correspond to a power of 90%. Considering a dropout rate of 10%, 440 patients in total were enrolled in the study.

Primary endpoint

The primary endpoint is change in Hemoglobin (Hb) from baseline to 2 weeks of medication follow-up.

Secondary endpoints

- Change in the Reticulocyte percentage (Ret%) after 2 weeks of treatment.
- Increase in Hemoglobin after 4 weeks of medication follow-up.
- Increase in Serum Ferritin after 8 weeks of treatment.
- Adverse events.
- Exploratory outcomes include MCV, MCH, and MCHC every 2 weeks at all time points and serum iron, TFs, TIBC at 8 weeks.

Adverse Event

Adverse events (AEs) refer to unfavorable medical events, which occur during clinical trials, whether the events are relevant to the trial or not. The investigators should follow up the adverse events until they disappear or stabilize and analysis the pathogens to determine the relationship between adverse event and research drug, which can be divided into: certainly related, possibly related, possibly unrelated, and certainly irrelevant. All adverse events that occur during the study must be reported in the adverse event table.

Classification of relation between adverse events and drugs

	Have Temporal relation with Research	Conform to the types of adverse effects	Adverse events alleviate after discontinuation	Adverse events present again after dosing	Situation can't be explained by progression

	drug				and treatment
certainly related	+	+	+	+	+
possibly related	+	+	+	/	+
certainly irrelevant	-	-	-	-	-
possibly unrelated	-	-	/	/	/

- Note,+ positive ； - negative；/, uncertain.

Collections of Adverse Events

	Intervention Group	Control Group
Adverse Event	N，（%）	N，（%）
All adverse events		
Adverse event related to research drug		
Serious Adverse event		
All serious adverse events		
Adverse event related to research drug		
Death		

Serious Adverse Event, SAE

SAE is adverse events that resulted in death, life-threatening situation, hospitalization, lengthening the time of hospitalization, disability, congenital abnormalities or birth defects. Patients are advised to return to the hospital in time or go to local hospital for treatment. The primary investigator or other participants should be present when patients occurred SAE. If necessary, the trail should be stopped immediately. Primary investigator should report the events to corresponding administrative department or the ethics committee of institution within 24 hours. The presentation of serious adverse events should use standard form provided by SFDA.

Statistical analysis

Full Analysis Set, FAS

Analysis of primary endpoint and secondary endpoint are following the basic principles of intention to Treat (ITT) approach to define the full analysis set. All randomized patients will be included in full analysis set for analysis. The missing data of efficacy evaluation will be estimated by last observation carried forward (LOCF).

Safety Set, SS

Patients who take research drug once should be included in safety set. The Safety Population is used for the analysis of safety, including adverse events, toxicity and laboratory evaluations.

Statistical Analysis

Baseline characteristics and summary statistics will be described by treatment group. Continuous variables will be summarized using descriptive statics, such as numbers of subjects, means with standard deviations, medians with interquartile ranges for non-normal data, and categorical data by counts and proportions. Qualitative variables will be summarized by frequency and percentage. Results with normal distributions are presented as mean values (±SD). Comparisons of ages, the baseline of Complete Blood Count and iron metabolism parameters between the study groups are performed with a t-test. Comparisons of genders and the incidence of adverse reactions between the two groups, which do not subject to normal distributions, will base on a X2 test. Confidence intervals (95%) for the difference of the changes in Hb level between two groups were calculated at each time point, and the equivalence was evaluated using the predefined margins of equivalence (±10g/L). Efficacy variables were analyzed on an intention-to-treat. If patients drop out, missing data were imputed by Last-Observation-Carried-Forward (LOCF) method. For the analysis of adverse events, patients who accepted oral iron tablets were included in the safety population. The threshold of a statistical significance is set as a P-value smaller

than 0.05. All tests were performed using STATA version 11.0.

Case distribution

This part includes summarizing how many patients enroll and complete the trial, describing the number of safety and valid analytical data set, and listing the data.

Case distribution（Unit：Case）

	Intervention Group	Control Group	Total
Enrollment			
FAS			
PPS			
SS			

Enrollment and missing number（Unit：Case）

	Intervention Group （N,%）	Control Group （N,%）
Enrollment Number		
Number of Completing treatment		
Number of missing		
Inclusion inconformity		
Violation of research plan		
Refuse to treat		
Loss of follow up		
Death		

Number of completing treatment（Unit：case）

	**Intervention Group**	**Control Group**	**Total1**
2 weeks			
4 weeks			
6 weeks			
8 weeks			

Comparison of demographics and baseline

Variables	Intervention Group	Control Group	P value
Gender<br>M/F			

Age（years）
WBC(×10^9/L)
RBC(×10^12/L)
PLT(×10^9/L)
MCV (fl)
MCHC(g/L)
MCH (pg)
SF
TIBC（%）
Clinical symptom (N, %)
Yes
No

Dosing Compliance

The box and aluminum cardboard of medication packages need return to investigators when follow up. Patients should be emphasized to return the entire pharmaceutical package, including the one remaining pharmaceutical packages, which can help researchers to calculate the information of dosing. Patients who took vitamin C also needed to return drug bottle every two weeks, which can help us to determine how much drug was available. Dosing compliance is defined as the ratio of actual dosage to the standard dosage. Actual dosage must within ±20% of the standard. Chi-square test will be performed to compare the differences in compliance between two groups.

Evaluation of CBC

Variables	Intervention Group	Control Group	P value
Hb$_0$(g/L)			
Hb$_2$(g/L)			
Hb$_4$(g/L)			
Hb$_6$(g/L)			
Hb$_8$(g/L)			
MCV$_0$(fl)			
MCV$_2$(fl)			
MCV$_4$(fl)			
MCV$_6$(fl)			
MCV$_8$(fl)			
MCHC$_0$(g/L)			

$MCHC_2$(g/L)			
$MCHC_4$(g/L)			
$MCHC_6$(g/L)			
$MCHC_8$(g/L)			
MCH_0(pg)			
MCH_2(pg)			
MCH_4(pg)			
MCH_6(pg)			
MCH_8(pg)			

Note, Hb_0, Hemoglobin level of baseline. Hb_2, hemoglobin level after 2 weeks. Hb_4, hemoglobin level after 4 weeks. Hb_6, hemoglobin level after 6 weeks. Hb_8, hemoglobin level after 8 weeks. MCV_0, MCV level of baseline. MCV_2, MCV level after 2 weeks. MCV_4, MCV level after 4 weeks. MCV_6, MCV level after 6 weeks. MCV_8, MCV level after 8 weeks. MCH_0, MCH level of baseline. MCH_2, MCH level after 2 weeks. MCH_4, MCH level after 4 weeks. MCH_6, MCH level after 6 weeks. MCH_8, MCH level after 8 weeks. $MCHC_0$, MCHC level of baseline. $MCHC_2$, MCHC level after 2 weeks. $MCHC_4$, MCHC level after 4 weeks. $MCHC_6$, MCHC level after 6 weeks. $MCHC_8$, MCHC level at 8 weeks.

Evaluation of iron metabolism indexes

Variables	Intervention Group	Control Group	P value
SF0(μg/ml)			
SF8(μg/ml)			
TS0(%)			
TS8(%)			
TIBC0(%)			
TIBC8(%)			

Note: SF_0, serum ferritin at baseline; SF_8, serum ferritin after 8 weeks; TS_0, transferrin saturation of baseline; TS_8, transferrin saturation after 8 weeks' time. $TIBC_0$, total iron binding capacity at baseline; $TIBC_8$, total iron binding capacity after 8 weeks.

Evaluation of adverse effects

Variables	Intervention Group	Control Group	P value
Incidence of adverse effects after 2weeks			
Classification and Severity after 2 weeks			
Nausea (N, %)			
Vomiting (N, %)			
Abdominal pain (N, %)			
Diarrhea (N, %)			

Incidence of adverse effects after 4 weeks
Classification and Severity after 4 weeks
Nausea (N, %)
Vomiting (N, %)
Abdominal pain (N, %)
Diarrhea (N, %)
Incidence of adverse effects after 6 weeks
Classification and Severity after 6 weeks
Nausea (N, %)
Vomiting (N, %)
Abdominal pain (N, %)
Diarrhea (N, %)
Incidence of adverse effects after 8 weeks
Classification and Severity after 8 weeks
Nausea (N, %)
Vomiting (N, %)
Abdominal pain (N, %)
Diarrhea (N, %)

Reference

1. Brugnara C. A hematologic "gold standard" for iron-deficient states? *Clin. Chem.* Jul 2002;48(7):981-982.

2. Camaschella C. Iron-deficiency anemia. *N. Engl. J. Med.* May 7 2015;372(19):1832-1843.

3. Moretti D, Goede JS, Zeder C, et al. Oral iron supplements increase hepcidin and decrease iron absorption from daily or twice-daily doses in iron-depleted young women. *Blood.* Oct 22 2015;126(17):1981-1989.

4. By Ronald Hoffman EJB, Jr., Leslie E. Silberstein, Helen Heslop, John Anastasi, Jeffrey Weitz. *Hematology: Basic Principles and Practice.* .

: Elsevier Health Sciences; 2017.

5. Sayers MH, Lynch SR, Charlton RW, Bothwell TH, Walker RB, Mayet F. Iron absorption from rice meals cooked with fortified salt containing ferrous sulphate and ascorbic acid. *Br. J. Nutr.* May 1974;31(3):367-375.

6. Cook JD, Monsen ER. Vitamin C, the common cold, and iron absorption. *Am. J. Clin. Nutr.* Feb 1977;30(2):235-241.

7. Cook JD, Reddy MB. Effect of ascorbic acid intake on nonheme-iron absorption from a complete diet. *Am. J. Clin. Nutr.* Jan 2001;73(1):93-98.

8. Hallberg L, Brune M, Rossander L. Effect of ascorbic acid on iron absorption from

different types of meals. Studies with ascorbic-acid-rich foods and synthetic ascorbic acid given in different amounts with different meals. *Hum. Nutr. Appl. Nutr.* Apr 1986;40(2):97-113.

9. Rocha DD, Capanema FD, Netto MP, de Almeida CAN, Franceschini SDC, Lamounier JA. Effectiveness of fortification of drinking water with iron and vitamin C in the reduction of anemia and improvement of nutritional status in children attending day-care centers in Belo Horizonte, Brazil. *Food and Nutrition Bulletin.* Dec 2011;32(4):340-346.

10. Hunt JR, Gallagher SK, Johnson LK. Effect of ascorbic acid on apparent iron absorption by women with low iron stores. *Am. J. Clin. Nutr.* Jun 1994;59(6):1381-1385.

11. Sadowski ZP, Alexander JH, Skrabucha B, et al. Multicenter randomized trial and a systematic overview of lidocaine in acute myocardial infarction. *Am. Heart J.* May 1999;137(5):792-798.
