# Supplementary material for: The Efficacy and Safety of Vitamin C for Iron Supplementation in Adult Patients With Iron Deficiency Anemia: A Randomized Clinical Trial
Source: JAMA Netw Open. 2020 Nov 2;3(11):e2023644. doi: 10.1001/jamanetworkopen.2020.23644 (PMC7607440; doi:10.1001/jamanetworkopen.2020.23644)
Supplement: Supplement 3. — Data Sharing Statement [file jamanetwopen-e2023644-s003.pdf]

Data Sharing Statement

Li. The Efficacy and Safety of Vitamin C for Iron Supplementation in Adult Patients With Iron Deficiency Anemia. *JAMA Netw Open*. Published November 02, 2020. 10.1001/jamanetworkopen.2020.23644

Data
Data available: No

Additional Information
Explanation for why data not available: The raw, processed data required to reproduce these findings cannot be shared at this time because the data also form part of an ongoing study. You may contact the authors at wangxiaoqin@shmu.edu.cn for more information.
